# Micronutrient Fortification of Food in Southeast Asia: Recommendations from an Expert Workshop

**DOI:** 10.3390/nu7010646

**Published:** 2015-01-19

**Authors:** Justine Gayer, Geoffry Smith

**Affiliations:** 1 International Life Sciences Institute (ILSI) Southeast Asia Region, 9 Mohamed Sultan Road, #02-01, 238959 Singapore; 2 Essential Micronutrients Foundation, 3 Pickering Street, #02-36 Nankin Row, China Square Central, 048660 Singapore; E-Mail: geoff.smith@emfglobal.org

**Keywords:** micronutrient deficiencies, fortification, public health strategy, Southeast Asia, policy

## Abstract

Micronutrient deficiencies remain a significant public health issue in Southeast Asia, particularly in vulnerable populations, such as women of reproductive age and young children. An important nutrition-specific intervention to address micronutrient malnutrition is fortification of staple foods and condiments. In October 2013, the International Life Sciences Institute (ILSI) Southeast Asia Region held a workshop on micronutrient fortification of food in Bangkok, Thailand. The objective was to engage multiple stakeholders in a discussion on food fortification and its importance as a public health intervention in Southeast Asia, and to identify and address key challenges/gaps in and potential opportunities for fortification of foods in ASEAN countries. Key challenges that were identified include: “scaling up” and mobilizing sustainable support for fortification programs in the form of multi-stakeholder partnerships, effecting policy change to support mandatory fortification, long-term monitoring of the programs’ compliance and efficacy in light of limited resources, and increasing awareness and uptake of fortified products through social marketing campaigns. Future actions recommended include the development of terms of engagement and governance for multi-stakeholder partnerships, moving towards a sustainable business model and more extensive monitoring, both for effectiveness and efficacy and for enforcement of fortification legislation.

## 1. Introduction

Micronutrient malnutrition is a global public health issue affecting 2 billion people worldwide, particularly pregnant women and young children. With the most widespread deficiencies being iron, zinc, vitamin A, iodine and folate, this “hidden hunger” contributes significantly to maternal and child morbidity and mortality [1,2], and is estimated to result in a 2%–3% loss in GDP [3].

Despite steady economic growth and an increase in food supply and subsequent energy intake over the past decade in Southeast Asia, micronutrient deficiencies continue to affect women of reproductive age and children in a number of countries, with varying degrees of severity [4]. A survey of women of reproductive age and young children conducted in 2010 in Vietnam indicated that a large proportion of the population were still at risk for vitamin A, vitamin B_12_, folate and zinc deficiency [5]. Recent data from the South East Asian Nutrition Survey (SEANUTS) [6,7,8,9], a four-country, nationally-representative study of 16,744 children aged 6 months to 12 years, showed moderate to severe levels of anemia in children in the youngest age groups and those children from rural areas, and borderline serum retinol levels in a large percentage of children across all four countries. In addition, emerging deficiencies in other micronutrients such as vitamin D are also prevalent in children in all four countries covered in the SEANUTS studies [6,7,8,9].

Fortification of staple foods and condiments is considered to be a cost-effective strategy to address the current nutrient gap experienced by the lowest socioeconomic groups, caused by the inability to afford a diversified diet [10]. Food fortification has garnered increasing interest and advocacy as an important micronutrient-related intervention both globally and in Southeast Asia [11,12,13,14]. The Copenhagen Consensus on Hunger and Malnutrition held in 2012 ranked food fortification among the top three international development priorities [11]. Four ASEAN region countries, namely Indonesia, Lao PDR, Myanmar and Vietnam, have joined the Scaling Up Nutrition (SUN) movement, a global network of stakeholders committed to elevating nutrition on the international agenda [12]. The SUN movement lists fortification of foods as a “Specific Action for Nutrition”—part of a multi-intervention approach to address malnutrition [12]. In addition, the “Sustainable Micronutrient Interventions to Control Deficiencies and Improve Nutritional Status and General Health in Asia” or SMILING Project, a collaborative project between research institutions from Cambodia, Indonesia, Lao PDR, Thailand, and Vietnam, and European partners, has identified food fortification as one of the “priority interventions” to alleviate micronutrient malnutrition in Southeast Asia [13].

In October 2013, the International Life Sciences Institute (ILSI) Southeast Asia Region, a non-profit scientific organization, held a two-day regional workshop on Micronutrient Fortification of Foods, in Bangkok, Thailand, with the support of its Taskforce on Fortification and in cooperation with the Food Science and Technology Association of Thailand (FoSTAT). The purpose of the workshop was to bring together multiple stakeholders to identify and address key challenges/gaps in and potential opportunities for micronutrient fortification of foods as a public health strategy in Southeast Asia, with contributions from academia, public health officials and industry. The conference agenda and presentations are available at the ILSI SEA Region website [14]. This report shares the authors’ views of key insights from the workshop and discusses implications for nutrition program planning, policy and future outlook in regard to food fortification in Southeast Asia.

## 2. Materials and Methods

Workshop participants were experts in the field of nutrition, public health and micronutrient fortification programming from across Asia, representing academia, government, non-governmental organizations and food industry. The three key areas of discussion in the workshop were around public-private partnerships in micronutrient fortification of foods; regulatory status and framework for micronutrient fortification; and monitoring and evaluation of food fortification programs. The following questions were posed to workshop participants:

(a) Public-private partnerships
  - How can the private sector help to support micronutrient fortification programs in Southeast Asia?
  - What are the current challenges involved in developing public-private partnerships?
  - What are recommended strategies to facilitate public-private partnerships in the Southeast Asia region?
(b) Regulatory status
  - What is the current regulatory status of fortification in Southeast Asia?
  - What are the differences in approach between mandatory and voluntary fortification including considerations when deciding which one to implement?
  - What are the challenges involved in implementing regulations pertaining to food fortification in Southeast Asia?
(c) Monitoring and evaluation
  - What are the key monitoring and evaluation measures that are in place for food fortification programs in Southeast Asia?
  - Where are the gaps in monitoring and evaluation of existing food fortification programs in Southeast Asia?
  - What are recommended additional monitoring and evaluation that should be undertaken/can be reasonably achieved in Southeast Asian countries?

## 3. Results

### 3.1. Public-Private Partnerships in Food Fortification

In Southeast Asia, the private sector has a huge potential to contribute to food fortification as a public health strategy, helping the public sector to solve some of the issues surrounding malnutrition by making fortified products available and affordable or bringing new products/strategies around nutritious foods to Southeast Asian countries whilst aligning with their respective national nutrition plans.

#### 3.1.1. Role of Private Sector in Fortification Programs in Southeast Asia

Private sector engagement is pivotal in the scaling up of food fortification programs to provide cost support for production and potentially financial and technical support towards joint research efforts into efficacy and effectiveness of the fortification program.

The private sector can provide food technology expertise including technical capability in the addition of fortificants to foods, helping to ensure minimum impact to sensory characteristics and stability of the product, whilst establishing ongoing monitoring of product quality.

Market and consumer insights that industry possesses can aid in identifying the most appropriate food vehicle, affordable price, packaging and positioning of the product with consumers. The private sector has access to wide distribution networks and logistics expertise which could ultimately lead to increasing availability and affordability of fortified products.

One of the key issues in fortification is accurate estimations of intakes of the fortified (or potentially fortified) foods. Generally this is done based on government food intake surveys, or on food balance sheets often using FAO data. However, this data is not always up to date, and food balance sheets do not provide clear estimates of individual or even household consumption of fortified foods. A potential role of public-private partnerships could be strengthening food intake data collections, and possible use of private data combined with public data to achieve more accurate estimates.

Industry has the capability to mobilize multiple communications channels including mass media and social networks which are critical in reaching the target population, in order to create public awareness of the micronutrient deficiency problem and demand for the fortified product.

Multi-stakeholder partnerships across a number of levels—global, regional, country, community—are crucial for effective and sustainable food fortification programs [15].

#### 3.1.2. Challenges in Developing Public-Private Partnerships in Southeast Asia

There exist a number of challenges in developing multi-stakeholder partnerships for food fortification in Southeast Asia. There is sometimes a perceived conflict of interest between the public and private sector with the seemingly incompatible priorities of promoting public health and returning a profit to shareholders. Workshop participants highlighted an element of distrust among some potential collaborators in the region.

There must be mutual benefit derived from participating in public-private partnerships, with the private sector unwilling to take a risk if the market is not viable from a business perspective. Production costs and price to the consumer must be balanced so as not to discourage supply or demand.

Trade barriers may exist across borders in terms of differing fortification levels permitted and labeling declarations, which may prove restrictive to companies selling products in multiple countries in Southeast Asia. This is particularly relevant once the ASEAN free trade agreement comes into effect in 2015 and open trade is put in place. In addition, industry may be frustrated by the relative slowness and level of bureaucracy experienced in collaborating with the public sector.

Many small to medium enterprises in Southeast Asia may not have sufficient knowledge or understanding of how to contribute to the reduction and prevention of malnutrition in their respective countries. They may not have sufficient funds to contribute to fortification programs or be risk averse towards such an investment. The market situation for the specific food vehicle may be fragmented and various industry players difficult to coordinate and mobilize.

#### 3.1.3. Facilitating Public-Private Partnerships

A number of recommendations were made on how to facilitate effective multi-stakeholder partnerships in Southeast Asia. A clear mechanism on how to engage private sector partners in the ASEAN region is needed. This framework and guiding principles for public-private partnerships in food fortification would include who should initiate such partnerships, the terms of engagement and governance, including dealing with potential conflicts of interest, and promoting transparency, accountability and equity amongst all stakeholders. There is a need to build trust among all parties.

In order for public-private partnerships to be successful, food fortification as a public health measure should move away from corporate social responsibility (CSR) to an effective business model. CSR activities have an important place, but fortification aims to reach large populations, and CSR efforts are usually too limited in scope. Feasibility studies and investment plans as part of an overall “stakeholder awareness” program should be developed and the costs of fortification outlined. Fortification of staple food was reported to add less than 1%–2% to the cost of the food, and an even lower percentage for packaged foods (depending on the extent and range of fortificants added). With mandatory fortification, the costs for all food producers are equal so no individual company is at a competitive disadvantage. Feasibility studies have been successful in demonstrating the benefit of fortification to the public and private sectors, for example in the development of large scale fortification of vegetable oil with vitamin A in Indonesia [16].

Innovative partnerships should be encouraged that promote mutual benefit and provide industry with a point of difference or additional competitive advantage, making it more attractive for other industry partners to join. There are some successful public-private partnerships already in Southeast Asia, with organizations such as the Global Alliance for Improved Nutrition (GAIN) working with governments, other NGOs and the private sector on food fortification programs over a number of years. Success stories in public-private partnerships already operational in the ASEAN region should be shared in further public forums. Case studies from other regions where the private sector has contributed to public health strategy should be highlighted. For example, in Canada the private sector adopted folic acid fortification of wheat flour in 1997, prior to mandatory fortification regulations which were enacted in Canada in 1998 (and in part motivated by the market in the US where mandatory fortification had already been introduced). Already in 1997 reductions in the incidence of neural tube defects in Canada could be seen, and the establishment of mandatory fortification has resulted in an even more significant reduction in the incidence of neural tube defects [17].

Finally, the stakeholders commonly referred to in public-private partnerships should be expanded to include Civil Society Organizations (CSOs) including international and national non-governmental organizations, community-based organizations, women’s groups, advocacy groups and social movements. These organizations provide a diverse range of expertise in areas such as research and social marketing, and a broad reach, particularly at the grass-roots level, that can greatly enhance public-private partnerships in food fortification programs.

### 3.2. Regulatory Status and Legal Framework for Food Fortification in Southeast Asia

The legal framework is imperative to the successful implementation of large scale food fortification programs [18].

#### 3.2.1. Regulatory Status of Micronutrient Fortification in Southeast Asia

Regulatory status of micronutrient fortification of foods in Southeast Asia varies greatly between countries. Results from a survey on regulatory status of micronutrient fortification in 10 ASEAN countries [19] presented at the workshop showed that voluntary fortification with vitamins and minerals is permitted in most countries in Southeast Asia, with considerable differences in approach in regulating, such as the food vehicle, micronutrient form, minimum and maximum levels and claims permitted. Mandatory fortification of salt with iodine is present in 8 out of 10 ASEAN countries, and mandatory flour fortification with iron is in place in Indonesia and the Philippines [19]. Fortification of sugar and cooking oil with vitamin A is mandatory in the Philippines, and fortification of unbranded cooking oil with vitamin A will be mandatory in Indonesia from early 2015 onwards. Table 1 presents a summary of mandatory fortification status in ASEAN countries.

**Table 1:** Mandatory nutrient fortification in ASEAN [19].

Nutrient	Food Vehicle	Country
Iodine	Salt	All 10 countries, except Brunei, Singapore and some parts of Malaysia
Iron	Wheat flour	Indonesia
	Wheat flour and rice	Philippines
Vitamin A	Condensed, evaporated and filled milk; margarine	Malaysia
	Wheat flour, refined sugar, cooking oil	Philippines
	Condensed milk, margarine	Thailand
	Unbranded cooking oil	Indonesia (commencing 2015)
Vitamin D	Margarine	Malaysia
Folic acid and B vitamins	Wheat flour	Indonesia
	Rice	Thailand
Zinc	Wheat flour	Indonesia

#### 3.2.2. Challenges in Regulation of Food Fortification in Southeast Asia

Both voluntary and mandatory fortification present challenges in Southeast Asia. With voluntary fortification, the effectiveness of the program relies on the private sector to first adopt fortification, which may permit fast action, but in terms of effectiveness the impact may be reduced as the need for education may delay the broad roll-out of fortified foods, as not all industry partners may be willing to fortify.

Voluntary fortification relies on industry choosing the food vehicle to fortify, which may result in the promotion of a fortified foods not widely consumed by the target population, thereby not achieving adequate coverage. Even for mandatory fortification, adequate coverage is an issue, and some countries in Southeast Asia have been reported to be fortifying three separate commodities with vitamin A. There is a potential risk of exceeding recommended levels of intake, but whether this is true for any population sub-groups in Southeast Asia has not been established. Questions about exceeding the tolerable upper intake level (UL) for micronutrient intake through fortified foods, particularly for the youngest subgroup, children aged 2 to 8 years, have been raised in the US for folic acid, niacin, and zinc, examined in a recent review by Berner *et al* [20]. However, as noted in the review, “the intakes might not be truly of public health concern if the UL established for children are set too low. Questions about the quantification of the UL remain because of lack of evidence of any adverse effects, even though many children have usual intakes above the UL for nutrients such as zinc; because of a lack of data on specific hazard identification relevant to children; and because the extrapolation of adult UL values to children on the basis of body weight is controversial and can be fraught with error”. Since there are far fewer fortified foods in Southeast Asia this may not yet be a concern, but must be evaluated in future fortification proposals.

Without proper standards, there is no legal recourse with voluntary fortification to ensure that fortification levels meet the published standards, which may result in industry fortifying at lower levels than specified in order to save cost, thereby reducing the ability of the fortification program to impact the micronutrient status of the target population. However once standards are established (e.g., 15% of the RDA) then normal enforcement mechanisms kick in to ensure fortification levels.

In a number of countries in Southeast Asia where mandatory fortification is in place, proper enforcement is sub-optimal and still presents a challenge. There is a need to make more widely available tools to allow QA to be conducted in the field to assist in effective enforcement of food fortification legislation. The development and implementation of mandatory fortification is in itself a lengthy process, requiring interim measures, such as voluntary fortification, to be put in place.

In both voluntary and mandatory fortification, barriers to trade may exist where a country sets a standard purely on domestic requirements and subsequently products are unable to be exported. The capacity to monitor and enforce legislation may also be affected by exporting to other countries with the region.

#### 3.2.3. Recommendations: Regulation of Food Fortification in Southeast Asia

Trade across borders in the ASEAN region should be taken into account at the development stage of food fortification strategies and standards. It was recommended that a forum be initiated for discussion of a common ASEAN region standard for adding micronutrients to foods for the nutritional benefit of the population, using WHO/FAO recommendations as a basis. At present, most of the countries fortifying flour in the ASEAN region are not fully harmonized with the WHO/FAO guidelines [21].

Five key priorities were proposed for policy makers when considering mandatory fortification, which included:

- adequately defining the public health objective to be addressed by fortification;
- strengthening the evidence base for all policy options being considered, including improving national data on micronutrient status;
- assembling relevant data on the economic impact of micronutrient deficiencies and costs of various strategies to address these;
- using social marketing as a complementary strategy to food fortification;
- establishing an effective monitoring and evaluation system for mandatory fortification for compliance/enforcement and to measure if the specified public health objective is being met.

Significant political will from all stakeholders is required for the support of mandatory fortification, although this can be achieved as this strategy has been used effectively for more than 80 years in various countries. It was recommended that fortification be included as part of a national multi-intervention strategy towards micronutrient deficiency prevention and control. This national strategy has been adopted in Indonesia, Vietnam and Myanmar. These three countries, along with Lao PDR, have joined the Scaling up Nutrition network and as part of their commitment have incorporated food fortification into their national nutrition plans as a “specific nutrition intervention with proven effectiveness”. Vietnam’s “National Guidelines on Micronutrient Deficiencies Control” and the Myanmar “National Plan of Action for Food and Nutrition 2011–2015” address food fortification as a strategy to reduce micronutrient deficiency.

A more effective regulatory monitoring system for food fortification is necessary in Southeast Asian countries both to monitor health and micronutrient status of target populations and compliance with fortification regulations.

### 3.3. Monitoring and Evaluation of Fortification Programs

#### 3.3.1. Key Monitoring and Evaluation Measures in Southeast Asia

There exist some monitoring systems in place in Southeast Asia for food fortification programs. Portable technical equipment is now available to allow compliance testing in the field, measuring levels of fortificant contained in the food product (for example iron in fish sauce, vitamin A in cooking oil and iodine in salt in Indonesia). Despite being identified as a promising tool for monitoring fortification programs [22,23,24], these tools must be used more broadly in Southeast Asia to be effective.

Monitoring of micronutrient status and compliance with fortification programs exists in some national health surveys in Southeast Asia, for example iodine status/intakes using Urinary Iodine Excretion (UIE) is integrated into the National Health Examination Survey in Thailand. In addition, both Vietnam and Myanmar have regular national micronutrient surveillance built into their national nutrition plans. However this is an area that needs urgent attention and consideration should be given to an ASEAN-wide agreement on protocols.

#### 3.3.2. Challenges and Gaps in Monitoring and Evaluation in Southeast Asia

Efficacy and effectiveness studies of food fortification programs may be used to justify the use of these interventions. Efficacy studies by their nature are evaluated under “best case” controlled circumstances, but effectiveness studies are difficult to monitor and complex to evaluate. It is difficult to assess the impact of food fortification in a national program where other interventions are implemented concurrently and especially when there are several fortified food products being consumed at the same time.

Good quality food consumption data is essential in both defining nutrient gaps to target with food fortification programs and to measure consumption of the fortified food, and is currently lacking in some countries in Southeast Asia. Work is currently being undertaken to investigate current methodologies used and challenges/limitations in obtaining this data in Southeast Asia. The food industry can potentially assist in improving food composition tables for processed foods—another role for public-private partnerships in food fortification. Food composition data is also critical for determining micronutrient intakes from unfortified foods, and steps to strengthen the process and regularity of the composition analysis of foods should be considered. For the region, these analyses are entered into the ASEANFOODS database maintained by Mahidol University under the INFOODS program administered by the FAO [25].

## 4. Discussion

The lessons learnt from the Workshop on Micronutrient Fortification of Food support and build on what is currently known about the issues surrounding food fortification in Southeast Asia, and have implications for nutrition program planning in the region.

The question is not whether there should be public-private partnerships in fortification in Southeast Asia, but rather how to engage in effective partnerships. Tools are currently being developed to assist in facilitating public-private partnerships. The SUN Business Network, as part of the Scaling Up Nutrition (SUN) Movement, has developed a “Private Sector Engagement Toolkit” to provide guidance in overcoming challenges in public-private partnerships, giving recommendations and identifying best practices [26]. This may be tailored to assist in establishing public-private partnerships in Southeast Asia. Non-governmental organizations such as GAIN already engage in such partnerships in the region. Created in 2002 at a Special Session of the UN General Assembly on Children, GAIN was tasked with the role of facilitating such partnerships and can provide significant expertise and best practice in this area. The ASEAN Economic Community (AEC) has developed guidelines on how to deal with the issues surrounding the movement of fortified and unfortified foods across borders, which may be used a reference in the development and implementation of food fortification programs in the region [27].

Much can be learned about multi-stakeholder partnerships and national intervention programs from case studies from the region. Major progress occurred in the Philippines in the 1990’s when government agencies, NGOs and industry came together to address micronutrient deficiency, launching the national salt iodization program in 1995, which later became a National Act. The Sangkap Pinoy Seal (SPS) was then developed as a seal of recognition appearing on the packaging of fortified foods. After reviewing the National Nutrition Survey of 1998, the Philippines government launched the Food Fortification Strategic Plan which encouraged public-private partnerships and resulted in the mandatory fortification a number of staple foods.

Food fortification now forms part of the national nutrition guidelines in Vietnam. Deficiencies of vitamin A, iron, zinc and iodine remain a public health issue in Vietnam, and although many programs have targeted these deficiencies, they were not well integrated or regulated. The Vietnamese government subsequently developed the “National Nutrition Strategies for 2010–2020”, with a focus on reduction of stunting and the prevention and control of micronutrient deficiencies. The “National Guidelines for Micronutrient Deficiencies Control”, developed as part of these national strategies, aims to increase coverage of programs to improve micronutrient status; develops standards for national programs and provides reference materials that aid health care professionals.

Investigation of the current regulatory framework in Southeast Asian countries also reveals critical elements shaping implementation of food fortification programs. A recent review of the legal framework for food fortification using examples from Vietnam and Indonesia showed several specific factors and components crucial to the success of fortification programs [18]. The legal framework should clearly state the specific public health objective that fortification is to address, and key indicators of whether this objective has been met should be incorporated into national health surveys. Monitoring and enforcement of compliance are also critical, with lack of facilities, lack of capacity and no clear line of responsibility for enforcement amongst the relevant authorities reportedly hampering fortification efforts in Vietnam, despite legal provisions being made [18].

Communicating the benefits of fortified foods and educating consumers on their use is also imperative to the success of food fortification programs [28]. Public, private and civil society organisations can provide complementary approaches to communication. The iron-fortified fish sauce (IFFS) project in Cambodia, undertaken by the Reproductive and Child Health Alliance (RACHA) in collaboration with the National Sub-committee for Food Fortification and financially supported by GAIN, included a comprehensive social marketing and advocacy campaign implemented at the grass-roots and national levels, including community cooking demonstrations and puppet shows. Political commitment from government and development partners as well as participation from the private sector, local authorities and communities in these social marketing campaigns played a critical role in the success of the program, but will need to be sustained to ensure awareness is raised from its current level [29].

Continued support for monitoring efficacy will also be critical for sustaining fortification programs [30], and public-private partnerships can be effective to measure fortification levels and fortified food intake levels. It is now time to collect more evidence on the impact of food fortification programs in Southeast Asia in regards to a reduction in morbidity and mortality, particularly in women of reproductive age and children. Health economic measurements will support advocacy efforts towards a reduction in micronutrient deficiency in Southeast Asia.

## 5. Conclusions

Food fortification is an effective strategy to address micronutrient deficiencies, which are still prevalent in some populations in Southeast Asia. Multi-stakeholder partnerships will be imperative to the implementation and sustainability of fortification programs, but will require an overall framework with clear guidelines for public, private and civil society collaboration in ASEAN countries. There is a need for more local nutrient intake data, not only to identify and quantify nutrient gaps, which is critical in defining the public health problem, but for evaluating the impact of fortification programs. This, in turn, will aid in gaining support from policy makers and other key stakeholders. Improved field support to monitor compliance and sustain social marketing efforts at the community level is also required in ASEAN countries with food fortification programs already in place.
